# Joint and individual associations between multiple vitamins and sperm quality in adult men

**DOI:** 10.3389/fnut.2025.1534309

**Published:** 2025-03-28

**Authors:** Wen Yao, Juan Zhang, Weihong Yan, Di Xie, Ping Tuo, Jie Liu, Xiaoling Zhao, Yiwen Xiong, Yang Li, Tiejun Pan

**Affiliations:** ^1^Reproductive Medicine Center, General Hospital of Central Theater Command, Wuhan, Hubei, China; ^2^Department of Urology Surgery, General Hospital of Central Theater Command, Wuhan, Hubei, China

**Keywords:** vitamins, sperm quality, DFI, serum, BKMR

## Abstract

**Background:**

Several studies have suggested that a healthy diet is associated with improved male fertility outcomes. However, the joint and individual associations between the status of multiple vitamins and sperm quality remain unclear.

**Objectives:**

This study aimed to investigate the associations between serum vitamin levels and sperm quality parameters in adult men.

**Methods:**

A cross-sectional study was conducted among 156 adult men seeking fertility care at a reproductive center from December 2023 to June 2024. Blood and semen were collected on the same day to determine the concentrations of nine kinds of vitamins (B1, B2, B6, B9, B12, C, A, D, E) and five parameters of sperm quality [total sperm number, sperm concentration, progressive motile sperm, morphologically normal rate, and DNA fragment index (DFI)]. The joint and individual associations between vitamin levels and sperm quality were analyzed using multiple linear regression and Bayesian kernel machine regression (BKMR) models.

**Results:**

Increased tertiles of vitamin B1 and 1,25-dihydroxyvitamin D3 [1,25–(OH)_2_-D_3_] in serum were associated with higher sperm quality (all *P* for trends < 0.10). Compared with the lowest tertiles, the highest tertiles of vitamin B12 had β: 3.0 (95% CI: 0.8, 5.2) increasing in DFI, and vitamin A was negatively associated with progressive sperm motility (*P* for trends = 0.05). We generally found null results between multiple vitamin levels and the parameters of sperm quality in the BKMR models.

**Discussion:**

These research findings imply that vitamins could have a dose-dependent dual effect on sperm quality. More specifically, the impact of vitamins varies according to their dosage levels and types. Personalized vitamin supplementation may be more effective than taking multivitamins in improving sperm quality.

## 1 Introduction

Globally, sperm concentration dropped by 51.6% between 1973 and 2018, with a steeper decline of 2.64% per year after 2000 (1). Correspondingly, there has been a significant increase in male infertility over the past two decades, bring depression, anxiety, stress, and a decreased quality of life to adult males (2, 3). Infertility affects 8%−15% of couples worldwide, with male factors accounting for 50% of cases (4). Therefore, identifying factors detrimental to sperm quality and implementing preventive measures to enhance male reproductive health is crucial. Despite some initiative programs have launched, male infertility persists due to unhealthy lifestyles and other factors environmental pollutants (5). Recent research confirms the significant role of a healthy diet in improving male fertility outcomes (6).

Vitamins, essential micronutrients in the human diet, play a vital role in normal physiological processes and various diseases (7). For male reproduction, previous studies have confirmed that vitamins have an impact on sperm quality (8). It is reported that vitamin B12 could increase sperm count, enhance sperm motility, and reduce sperm DNA damage through mitigating oxidative damage, optimizing energy production, and modulating inflammation response (9). Vitamin D receptors, crucial in the male reproductive tract, serve as predictive markers of sperm quality (10). Vitamin D may boost sperm motility by promoting ATP synthesis and increasing intracellular calcium ion concentrations (11). However, most studies narrow their focus on individual vitamins. Although some studies have shown that dietary supplements of vitamins can potentially boost semen production (12, 13), another study have indicated that there is no correlation between vitamins and sperm quality (14). Given these controversial results, the potential synergistic effects of multiple vitamins still require further research.

The issue of vitamins is a worldwide health problem. In the United States, 61% of children and adolescents are reported to suffer from vitamin D insufficiency, with 9% experiencing vitamin D deficiency (15). In India, 37 % of people had folic acid deficiency and 53 % had vitamin B12 deficiency (16). Understanding the relationship between vitamin levels and sperm quality is crucial for optimizing vitamin supplementation in clinical practice. To clarify the impact of vitamin levels on sperm quality, we determined the concentrations of nine kinds of vitamins (B1, B2, B6, B9, B12, C, A, D, E) in the serum and five parameters of sperm quality [total sperm number, sperm concentration, progressive sperm motility, morphologically normal rate, and sperm DNA fragment index (DFI)] in male adults to study the joint and individual associations between vitamin levels and sperm quality.

## 2 Materials and methods

### 2.1 Population

Participants included in this analysis were abstracted from the Environment and Male Reproduction Study, which is an ongoing cohort study aimed at investigating the relationship between environmental and dietary factors and male reproduction. Males aged over 20 years old and seeking fertility care at our Reproductive Center were eligible to enroll. Men with congenital malformation, trauma, or serious systemic diseases were excluded from the study. A total of 321 males were recruited from December 2023 to June 2024. Among them, 159 males measured the levels of vitamins. After excluding three males due to lack of sperm quality data, 156 males were included in the final analysis (Supplementary Figure S1). Informed consent was obtained from all subjects involved in the study. This study was approved by the Ethical Committee of the General Hospital of Central Theater Command (Number: [2023]087-01).

### 2.2 Covariates

Basic information was obtained by a questionnaire including age, ethnicity, height, weight, educational level, household income, smoking status, and drinking status. Ethnicity was classified into two categories: the Han nationality and other Chinese minorities (Dong, Hui, Gelao, Lisu, Mongolian, Tujia, and Zhuang nationalities). The smoking or drinking status was self-reported as never, current, or quit. Never refers to participants who have no history of smoking or drinking, current refers to participants who are smoking or drinking. Quitting refers to participants who had stopped smoking or drinking for 1 year (17). Body mass index (BMI) was calculated as weight/height^2^ (kg/m^2^).

### 2.3 Determination of vitamin level

Blood sampling was centrifuged at 2,000 × g and the supernatant was taken as a serum sample. Vitamin concentrations in serum were measured using an electrochemical method according to the manufacturer's instructions (LK3000VI, Lanbiao, Tianjin, China). Briefly, 20–80 μL serum was added to the processing solution (Lanbiao, Tianjin, China) and placed on the vitamin detector. Then, the polished electrode was inserted into the corresponding channel and activated. Vitamins were enriched and dissolved on the working electrode, and the levels of vitamins were determined according to the peak potential and peak current in the dissolution voltammetry curve. The level of 1,25-dihydroxyvitamin D3 [1,25–(OH)_2_-D_3_] in serum represents vitamin D status in humans.

### 2.4 Semen analysis

Semen samples were obtained by masturbation on the same day with blood drawing. Duration of abstinence was self-reported by participants, but the period was 2–7 days after sexual intercourse. The semen sample was weighed first to estimate its volume and liquefied at 37°C for 30 min. Then, the liquefied sample was mixed and 5 μL of the mixture was deposited on a cell counter. Sperm concentration, motility, and morphology were determined by the computer analysis system under a microscope (Olympus, Tokyo, Japan). Sperm motility was classified into four levels (A, B, C, D) according to the WHO criteria (18). Later, Papanicolaou staining was conducted for sperm smear and a total of 200 spermatozoa were counted to evaluate the sperm morphology with stricter criteria (19). The DFI was assessed by a sperm DNA fragmentation staining kit (Anke, Anhui, China). Briefly, a semen sample diluted to 5–10 million/ml was mixed with agarose and deposited 5 μL of the mixture on a slide. The slide was subsequently incubated in denaturation solution, lysis solution, washing solution, and ethanol. After Wright-Giemsa staining was performed, chromatin in the sperm head can be observed under microscope. Finally, DFI under the microscope was estimated as previously described (20):


$$\begin{array}{l} DFI\left(\%\right)=\frac{Fragmented+Degraded}{Total\;counted}\times 100 \end{array}$$

### 2.5 Statistical analysis

Continuous variables in baseline characteristics were presented as the mean ± standard deviation (SD), while categorical variables are shown as numbers and percentages (%). Missing data was removed from analyses. The associations between individual vitamin levels and sperm quality were evaluated using multiple linear regression models. Vitamin concentrations were log-transformed and categorized into tertiles to mitigate the bias introduced by skewed distribution and extreme values. Tests for trends were calculated by entering the tertiles of vitamins as ordinal categorical variables (1–3) in the linear models. Potential covariates in the models were selected by directed acyclic graph based on previous studies (Supplementary Figure S2) (21–27). We further excluded ethnicity and education level to avoid sparse data bias. Ultimately, age, BMI, household income, smoking status, and drinking status were included in the models. The restricted cubic spline (RCS) method was used to explore the potential non-linear relationships between serum vitamin levels and sperm quality after adjusting for confounding factors, including age, BMI, household income, smoking status, and drinking status. Crude associations and associations among normospermic males were calculated to assess the robustness of our findings.

We used Bayesian kernel machine regression (BKMR) models to estimate the joint and individual effects of multiple vitamins in a flexible and parsimonious manner (28). BKMR models, through their capacity to incorporate the correlations between multiple exposure variables and their ability to capture non-linear relationships, renders them extremely valuable to dissect how different nutrients interact to exert an influence on physiological processes. Considering that certain vitamins were significantly correlated in the Spearman correlation analysis (Supplementary Figure S3), we conducted a hierarchical variable selection with 50,000 iterations. While the first 25,000 iterations were dropped as burn-in phase. The groups were divided into water-soluble vitamins (B1, B2, B6, B9, B12, C) and fat-soluble vitamins (A, D, E). Covariates in the BKMR models were the same as in the linear models. Vitamin levels and continuous covariates (i.e., age and BMI) were centered and scaled for standardization. We used Gaussian distribution for the parameters of sperm quality in the kmbayes function. The group posterior inclusion probability (groupPIP) indicates the relative importance of each group in defining the associations between vitamins and sperm quality parameters. Meanwhile, the conditional posterior inclusion probability (condPIP) reflects the relative significance of individual vitamins in determining the associations between vitamins and sperm quality parameters within the group. All analyses were performed using R software [version 4.4.0, R Core Team (2024), R Foundation for Statistical Computing, Austria]. *P* < 0.05 was statistically significant, and *P* < 0.10 was considered as suggestively significant.

## 3 Results

### 3.1 Baseline information

The baseline characteristics of the study population are presented in Table 1. The mean age of the participants was 30.5 years old, and the mean BMI of the participants was 23.9 kg/m^2^. Among the males, 92.3% were Han nationality, 85.3% had a bachelor's or higher degree, 37.8% were current smokers, and 65.4% were current drinkers. As for the clinical characteristics, the mean total sperm number, sperm concentration, progressive sperm motility, morphologically normal sperm, and DFI were 683.8 million, 157.7 million per milliliter, 37.4%, 4.4%, and 14.6%, respectively. Compared with all males in the cohort, no differences were observed in basic information (Supplementary Table S1). The distribution of vitamins in serum is shown in Table 2. Vitamins were detected in all the samples.

**Table 1:** Characteristics of 156 males included in this study.

**Characteristics**	**Mean ± SD or *N* (%)**
**Basic information**	
Age (years)^a^	30.5 ± 3.3
Height (m)	1.7 ± 0.1
Weight (kg)	72.0 ± 8.7
BMI (kg/m^2^)^a^	23.9 ± 2.5
Duration of abstinence (day)	4.7 ± 1.8
**Ethnicity**	
Han	144 (92.3%)
Other Chinese minorities^b^	12 (7.7%)
**Education level**	
High school and below	23 (14.7%)
College and above	133 (85.3%)
**Household income (Yuan/month)** ^a^	
≤5,000 (reference)	11 (7.1%)
5,001–10,000	53 (34.0%)
≥10,001	92 (59.0%)
**Smoking status** ^a^	
Never (reference)	79 (50.6%)
Current	59 (37.8%)
Quit	18 (11.5%)
**Drinking status** ^a^	
Never (reference)	42 (26.9%)
Current	102 (65.4%)
Quit	12 (7.7%)
**Clinical characteristics**	
Total sperm number (mill)	683.8 ± 514.4
Sperm concentration (mill/ml)	157.7 ± 106.1
Progressive sperm motility (%)	37.4 ± 15.1
Morphologically normal (%)	4.4 ± 1.3
DFI(%)	14.6 ± 5.3

Nine males were missing for morphologically normal and 16 males were missing for DFI.

a Covariates included in the multivariable models.

b Other Chinese minorities included Dong, Hui, GeLao, LiSu, Mongolian, TuJia, and Zhuang nationalities.

BMI, body mass index; SD, standard deviation; DFI, DNA fragmentation index.

**Table 2:** Distribution of vitamin concentrations in serum among 156 adult males included in this study.

**Vitamins**	**Normal range**	**AM**	**GM**	**Percentile**		
				**25%**	**50%**	**75%**
Vitamin B1 (nmol/L)	70–180	69.5	68.2	60.0	70.5	78.0
Vitamin B2 (μg/L)	–	250.6	245.9	212.8	245.0	288.0
Vitamin B6 (μmol/L)	>20	19.3	18.6	15.4	18.7	22.3
Vitamin B9 (nmol/L)	>7	17.3	16.7	13.7	17.3	20.2
Vitamin B12 (pg/mL)	>203	380.7	362.7	295.0	350.0	448.5
Vitamin C (μmol/L)	>11	40.9	40.0	35.0	39.0	46.3
Vitamin A (μmol/L)	>0.7	0.6	0.6	0.5	0.6	0.7
1,25–(OH)_2_-D3 (nmol/L)	75–250	33.9	32.2	26.0	33.0	39.3
Vitamin E (μg/mL)	>5	12.3	12.2	11.0	12.0	13.0

The level of 1,25–(OH)_2_-D3 in serum represents vitamin D status in humans.

AM, arithmetic mean; GM, geometric mean.

### 3.2 Associations between individual vitamins in serum and sperm quality

**Figure 1:**
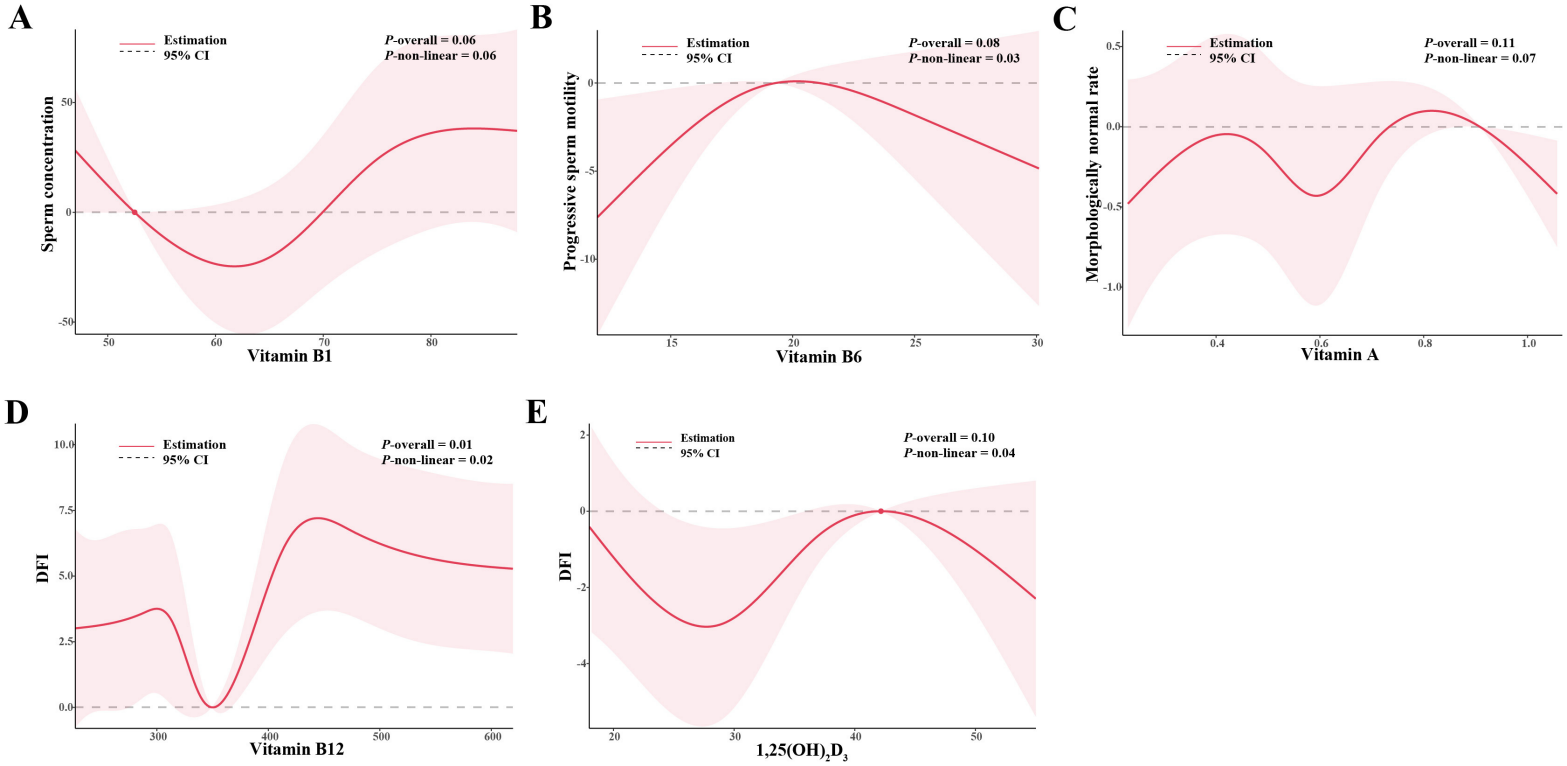
The non-linear associations between **(A)** vitamin B1 and sperm concentration, **(B)** vitamin B6 and progressive sperm motility, **(C)** vitamin A and morphologically normal, **(D)** vitamin B12 and DFI, **(E)** 1,25–(OH)_2_-D3 and DFI. Models were adjusted for age, BMI, household income, smoke status, and drinking status. BMI, body mass index; DFI, DNA fragmentation index.

The associations between vitamins in serum and sperm quality are presented in Table 3. Increased tertiles of vitamin B1 (>76.0 nmol/L) in serum were associated with higher total sperm number, sperm concentration, and DFI (all *P* for trends <0.10). Compared with the lowest tertiles (vitamin B12 < 311.6 pg/mL, 1,25–(OH)_2_-D_3_ < 28.6 nmol/L), the highest tertiles of vitamin B12 (>418.0 pg/mL) had β: 3.0 (95% CI: 0.8, 5.2) increasing in DFI, and 1,25–(OH)_2_-D_3_ (>37.0 nmol/L) had β: 0.5 (95% CI: 0.0, 1.0) increasing in morphologically normal rate. The highest tertile of vitamin A (>0.7 μmol/L) was inversely associated with progressive sperm motility compared to the lowest tertile (<0.5 μmol/L) (*P* for trends = 0.05). The results remained largely consistent in crude models and among normospermic males (Supplementary Tables S2, S3). Figure 1 showed the significant non-linear associations between serum vitamins and sperm quality using RCSs (All the non-linear associations were shown in Supplementary Figure S4). A non-linear, U-shaped relationship was observed between vitamin B1 and sperm concentration (*P* for non-linear = 0.06). Specifically, when the concentration of vitamin B1 elevated from the lowest to 60 nmol/L, the sperm concentration showed a decreasing trend. Nevertheless, when the vitamin B1 concentration exceeded 60 nmol/L, sperm concentration starts to increase. The total sperm number and vitamin B1 showed a similar trend (*P* for non-linear = 0.10). Regarding vitamin B6, when its concentration ranged from the lowest to 20 μmol/L, progressive sperm motility initially rose as vitamin B6 levels increased. As the concentration continued to rise above 20 μmol/L, progressive sperm motility starts to decline, suggesting a potential inverse U-shaped relationship (*P* for non-linear = 0.03). Additionally, irregular non-linear relationships were noted between vitamin A and morphologically normal rate, vitamin B12 and DFI, and 1,25–(OH)_2_-D_3_ and DFI (all the *P* for non-linear < 0.10).

**Table 3:** Associations between vitamin concentrations in serum and sperm quality among 156 adult males.

**Vitamins**	**Total sperm number**	**Sperm concentration**	**Progressive motile sperm**	**Morphologically normal rate**	**DFI**
	β **(95% CI)**	β **(95% CI)**	β **(95% CI)**	β **(95% CI)**	β **(95% CI)**
**Vitamin B1 (nmol/L)**					
Q1(<64.0)	Ref	Ref	Ref	Ref	Ref
Q2(64.0–76.0)	21.2 (−174.9, 217.3)	−6.9 (−47.7, 33.8)	−1.5 (−7.4, 4.4)	0.1 (−0.5, 0.6)	0.2 (−2.0, 2.4)
Q3(>76.0)	**243.0 (47.1, 438.9)**	**42.6 (1.9, 83.4)**	−3.0 (−8.9, 2.9)	0.1 (−0.4, 0.6)	1.9 (−0.3, 4.1)
*P* for trend	**0.01**	**0.04**	0.31	0.70	**0.09**
**Vitamin B2 (**μ**g/L)**					
Q1 (<225.6)	Ref	Ref	Ref	Ref	Ref
Q2 (225.6–278.0)	−33.2 (−236.4, 170.0)	5.4 (−36.8, 47.5)	3.1 (−2.8, 9.1)	−0.2 (−0.8, 0.3)	−1.2 (−3.5, 1.1)
Q3 (>278.0)	−32.5 (−232.8, 167.7)	0.5 (−41.0, 42.1)	2.38 (−3.5, 8.2)	−0.3 (−0.8, 0.2)	−1.3 (−3.5, 1.0)
P for trend	0.75	0.98	0.43	0.25	0.28
**Vitamin B6 (**μ**mol/L)**					
Q1 (<16.5)	Ref	Ref	Ref	Ref	Ref
Q2 (16.5–21.7)	−87.2 (−289.7, 115.4)	−15.5 (−57.5, 26.6)	4.7 (−1.3, 10.6)	0.1 (−0.4, 0.7)	0.3 (−2.0, 2.5)
Q3 (>21.7)	0.4 (−199.0, 199.7)	−15.8 (−57.2, 25.6)	4.1 (−1.8, 9.9)	0.1 (−0.4, 0.6)	−0.4 (−2.6, 1.8)
*P* for trend	0.97	0.46	0.18	0.66	0.71
**Vitamin B9 (nmol/L)**					
Q1 (<15.1)	Ref	Ref	Ref	Ref	Ref
Q2 (15.1–19.2)	−65.2 (−266.3, 135.9)	−2.4 (−44.1, 39.4)	3.4 (−2.6, 9.3)	−0.1 (−0.6, 0.5)	−0.3 (−2.6, 2.0)
Q3 (>19.2)	−95.7 (−296.5, 105.1)	−16.6 (−58.3, 25.1)	2.4 (−3.5, 8.3)	−0.2 (−0.7, 0.3)	−0.7 (−3.0, 1.5)
P for trend	0.35	0.43	0.42	0.46	0.53
**Vitamin B12 (pg/mL)**					
Q1 (<311.6)	Ref	Ref	Ref	Ref	Ref
Q2 (311.6–418.0)	−52.9 (−256.8, 151.0)	−33.6 (−75.5, 8.4)	1.7 (−4.3, 7.7)	−0.2 (−0.7, 0.4)	−0.9 (−3.1, 1.3)
Q3 (>418.0)	18.9 (−180.9, 218.7)	−2.7 (−43.8, 38.4)	0.1 (−5.7, 6.0)	0.0 (−0.6, 0.5)	**3.0 (0.8, 5.2)**
*P* for trend	0.86	0.89	0.96	0.92	**<0.01**
**Vitamin C (**μ**mol/L)**					
Q1 (<36.0)	Ref	Ref	Ref	Ref	Ref
Q2 (36.0–43.0)	5.7 (−200.6, 212.1)	−9.5 (−52.4, 33.3)	−0.3 (−6.4, 5.8)	−0.3 (−0.8, 0.3)	0.2 (−2.1, 2.5)
Q3 (>43.0)	58.0 (−150.4, 266.3)	−8.5 (−51.7, 34.8)	0.7 (−5.4, 6.8)	0.2 (−0.4, 0.7)	−1.3 (−3.6, 1.0)
*P* for trend	0.57	0.71	0.81	0.47	0.25
**Vitamin A (**μ**mol/L)**					
Q1 (<0.5)	Ref	Ref	Ref	Ref	Ref
Q2 (0.5–0.7)	−15.4 (−215.6, 184.9)	3.8 (−37.8, 45.3)	1.6 (−4.1, 7.4)	−0.3 (−0.8, 0.3)	−0.8 (−3.1, 1.5)
Q3 (>0.7)	−36.9 (−240.5, 166.7)	−3.7 (−45.9, 38.6)	**−5.9 (−11.8**, **−0.1)**	−0.1 (−0.6, 0.5)	0.7 (−1.6, 2.9)
*P* for trend	0.72	0.87	**0.05**	0.76	0.56
**1,25–(OH)**_2_ **-D3 (nmol/L)**					
Q1 (<28.6)	Ref	Ref	Ref	Ref	Ref
Q2 (28.6–37.0)	−16.8 (−222.4, 188.8)	−16.9 (−59.8, 26.0)	−2.3 (−8.4, 3.8)	0.1 (−0.5, 0.6)	0.8 (−1.5, 3.1)
Q3 (>37.0)	−142.8 (−335.0, 49.5)	−18.2 (−58.3, 21.9)	0.6 (−5.1, 6.3)	**0.5 (0.0, 1.0)**	1.1 (−1.1, 3.3)
*P* for trend	0.14	0.38	0.81	**0.07**	0.34
**Vitamin E (**μ**g/mL)**					
Q1 (<12.0)	Ref	Ref	Ref	Ref	Ref
Q2 (12.0–13.0)	86.9 (−122.6, 296.3)	12.3 (−31.0, 55.5)	−0.5 (−6.7, 5.7)	0.3 (−0.2, 0.9)	1.5 (−0.9, 3.8)
Q3 (>13.0)	64.1 (−140.4, 268.6)	29.9 (−12.3, 72.1)	1.5 (−4.5, 7.6)	0.4 (−0.2, 0.9)	1.61 (−0.7, 3.9)
*P* for trend	0.57	0.16	0.58	0.20	0.18

Linear regression models were adjusted for age, BMI, household income, smoke status, and drinking status. *P* for trend was derived by entering the quartiles of exposure as ordinal categorical variables (1–3) in the models. The level of 1,25–(OH)_2_-D3 in serum represents vitamin D status in humans.

Q1, Q2 and Q3 refer to the tertile (1–3) concentrations of the 156 volunteers in this study.

BMI, body mass index; DFI, DNA fragmentation index. The bold values indicated that there is a significant difference.

### 3.3 Associations of multiple vitamins in serum with sperm quality

**Figure 2:**
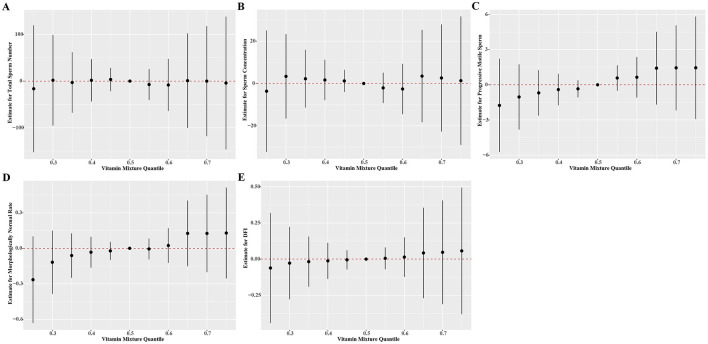
Estimate effects (95% credible intervals) of the mixtures of vitamins on **(A)** total sperm number, **(B)** sperm concentration, **(C)** progressive motile sperm, **(D)** morphologically normal rate, **(E)** DFI by BKMR models when all the vitamin concentrations at particular percentiles were compared to all the vitamins at their 50th percentile. The BKMR models were adjusted for age, BMI, household income, smoke status, and drinking status. Numerical values are presented in Supplementary Table S6. BMI, body mass index; BKMR, Bayesian kernel machine regression; DFI, DNA fragmentation index.

In the BKMR models, we found null results between multiple vitamin levels and the parameters of sperm quality (Figure 2, numeric results in Supplementary Tables S4). The fat-soluble vitamins appeared to have a higher contribution in defining the relationship between vitamins and sperm quality (Supplementary Tables S5). Consistent with the results in linear models, we also found that both total sperm number and sperm concentration exhibited a steadily increasing trend when the concentration of vitamin B1 elevated. There is also a unidirectional decline tendency of progressive sperm motility as the rising of vitamin A levels. However, we observed null association between vitamins and DFI within the measured concentration ranges of vitamins (Supplementary Figure S5).

## 4 Discussion

### 4.1 Main findings

Our study identified a non-linear relationship of vitamin B1 levels with total sperm number and sperm concentration (decreasing below 60 nmol/L and increasing above it) and an inverse U-shaped trend between vitamin B6 levels and progressive sperm motility (increasing below 20 μmol/L and decreasing above it). Additionally, 1,25–(OH)_2_-D_3_ and vitamin B12 showed irregularly but totally positive trend in morphologically normal rate and DFI, respectively. This suggests that vitamin B1, B12, and 1,25–(OH)_2_-D3 may play a crucial role in spermatogenesis and sperm DNA integrity. Conversely, vitamin A exhibited a unidirectional downward trend in progressive sperm motility as the concentration of vitamin A increased, indicating that excessive level of vitamin A may be deleterious to sperm motility.

The BKMR models, however, revealed null association between multiple vitamin levels and sperm quality parameters. This suggests that the beneficial effects of vitamins on sperm quality may be more complex than previously assumed. These findings highlight the importance of considering the dose-response relationship and potential interactions when interpreting the effects of vitamins on sperm quality.

### 4.2 Distribution of vitamins

Vitamins are a long-standing concern in human health. The recommended concentration of vitamin B1 for healthy individuals was 70–180 nmol/L (29). We found 71 (45.5%) and 88 (56.4%) participants were below the reference range of vitamin B1. Only 1 (0.6%) male under the definition level of vitamin B9 (7 nmol/L) and vitamin B12 (203 pg/mL) in serum (30, 31). There are 108(69.2%) subjects who were considered to have vitamin A deficiency because their serum level of vitamin A was below 0.7 μmol/L (32). The Endocrine Society proposed that 1,25–(OH)_2_-D3 level of 75–250 nmol/L was vitamin D sufficiency and 1,25–(OH)_2_-D3 level under 50 nmol/L was vitamin D deficiency (31). In this study, vitamin D deficiency was found in 141 (90.4%) individuals. No subjects exhibited deficiencies in vitamin B6 (<20 nmol/L) (33), vitamin C (<11 μmol/L) (34), or vitamin E deficiency (<5 μg/mL) (35). Additionally, there is currently no universally accepted cutoff value for vitamin B2 deficiency in serum (36). Our study revealed significant vitamin deficiencies among the participants, particularly in vitamin A and vitamin D. This is consistent with global trends and highlights the need for targeted interventions to improve vitamin status in the population (15, 37).

### 4.3 Comparison with previous studies

Vitamin D is the most focused vitamin in previous studies. In this study, we found that vitamin D was only associated with normal sperm morphology and had no correlation with other outcomes in multiple regression models and may have a non-linear relationship with DFI. Consistent with our results, a cohort study included 1,308 men reported that elevated serum 1,25–(OH)_2_-D3 was associated with a higher percentage of normal sperm morphology. They also found that total sperm count was increased with serum 1,25–(OH)_2_-D3 among participants with impaired semen quality (38). A study conducted in Iran reported no association between serum vitamin D levels and semen parameters in normospermic men (39). However, a cross-sectional study with 350 infertile men showed that higher serum vitamin D levels are positively associated with higher semen volume, sperm count, sperm total motility, and normal morphology rate (40). A prospective cohort study among 102 participants showed that vitamin D was not associated with parameters of sperm quality or DFI (41). As to animal models, vitamin D deficiency mice exhibited decreased motility and morphological abnormalities (42). And supplementation of 1,25–(OH)_2_-D3 significantly improved perfluorooctanesulfonate acid induced sperm quality decline and testicular damage (43). Vitamin D exerts both calcium-dependent and calcium-independent effects. This implies that certain effects of vitamin D can be mitigated by a calcium-rich diet, which is likely accessible to the majority of people (44, 45). Considering that calcium level was not measured in our study, the variation in calcium concentration might be a contributing factor to the divergent results. These discrepancies may also be attributed to differences in study populations, sample sizes, and statistical methods.

Our study also demonstrated positive associations between higher level of vitamin B1 and total sperm number, sperm concentration, and DFI in males. However, a study involving 210 men found an inverse association between vitamin B1 and semen normality (46). It is worth note that their data about vitamin B1 levels were acquired from the questionnaire and the participants were limited to unexplained or idiopathic infertility men. Mice with mutations in the high-affinity vitamin B1 transporter gene SLC19A2 are infertile with small testes and spermatogenesis arrested (47). The highest tertile of vitamin B12 and vitamin A showed a negative correlation with sperm quality in our results. Conversely, previous studies indicated that the levels of vitamin A in men with abnormal sperm parameters were either comparable to or lower than those of normospermic men (48, 49). Nevertheless, they classified sperm quality according to the 4th edition of the World Health Organization (WHO) laboratory manual, which has changed a lot now. Crude statistical analyses and small sample sizes also reduced the credibility of their results. Another study with 29 infertile men found a positive association between vitamin B9 and sperm parameters, while there is no association between vitamin B12 and sperm quality (20). The non-linear association, interaction effect, and sample size may contribute to those differences. Additionally, a multicenter randomized clinical trial involving 2,370 men reported that supplementing with folic acid did not improve semen quality (50). *In-vivo* studies in mice have demonstrated that nearly all vitamin deficiencies can result in the deterioration of sperm parameters (44, 51). Although some studies suggest that supplementing with some vitamins may improve sperm quality (12, 52, 53), null association was found in this study between the other vitamin levels and sperm quality.

### 4.4 Interpretation

The potential mechanisms underlying the associations between vitamins and sperm quality are complex and multifactorial. We found that vitamin B1, B6, B12, D, and A may have an impact on sperm quality. However, little is known about the mechanisms between vitamin B1 and sperm quality. Vitamin B1 undergoes phosphorylation to form thiamine diphosphate. This compound is essential for the oxidative catabolism of carbohydrates and the synthesis of ATP within mitochondria (54). We speculate that vitamin B1 exerts dual effects on sperm quality, which are attributed to alterations in mitochondrial energy metabolism and the oxidative stress response (55). Vitamin B6, B9, and B12 have been reported to play crucial roles in the carbon cycle and methylation processes. These vitamins participate in the methionine synthase reaction, a key step within the one-carbon metabolism pathway, to synthesize S-adenosylmethionine. This compound is then engaged in a vast array of methylation reactions, playing a crucial role in various biological processes (56, 57). Vitamin D receptor and associated metabolizing enzymes in the human male reproductive tract suggests a crucial role of vitamin D in spermatogenesis (58). Vitamin D deficiency and *Vdr*-null mouse models indicated that lipid metabolism, calcium imbalance, sex hormones, and DNA damage may mediate the effect of vitamin D on sperm quality (10, 42, 59). Vitamin D deficiency was also reported to disrupt the one-carbon cycle and transsulfuration pathway, increasing circulating homocysteine, thus affecting sperm quality (44). Vitamin A is also indispensable for spermatogenesis since most germ cells were arrested at the undifferentiated stage in vitamin A-deficient mice (60). It seems that vitamin A serves as a trigger of the meiosis process for spermatogonia. Almost all the vitamins are reported to act as antioxidants and reduce oxidative damage to maintain the integrity of the sperm DNA (61, 62). In addition to vitamin deficiency, excessive vitamins also need attention. Chronic vitamin A excess in mice could impair sperm motility and head morphology in mice (63). As a fat-soluble vitamin, vitamin D is absorbed and stored in the body's adipose tissues and liver. This characteristic is closely related to the risk of vitamin D toxicity, which may lead to hypercalcemia (64). While Ca^2^^+^ is a key second messenger in regulating sperm physiological processes like capacitation, hyperactivation, and the acrosome reaction (65). The detailed mechanism of vitamins in the spermatogenesis process is still unclear. Further research is needed to fully understand the mechanisms and dose-response relationships between vitamins and sperm parameters.

### 4.5 Strengths and limitations

The strength of our study lies in the use of BKMR methods to explore both joint and individual relationships between multiple vitamin levels and sperm quality. While the cross-sectional design precludes causal inference, the robust dose-response trends (e.g., *P* for trend = 0.05 for vitamin A and progressive sperm motility) strongly justify prospective cohort tracking vitamin levels and semen parameters over time. Additionally, we just measured the vitamin and sperm quality in one spot, which neglected the variations in vitamin levels and sperm analysis within individuals. The limited sample size also reduced the persuasiveness of our results. Larger prospective studies with multiple measurement points are warranted in the future. A previous study found inconsistent levels of 1,25–(OH)_2_-D3 in seminal plasma and serum, suggesting that seminal plasma vitamin D is involved in regulating sperm motility (66). Unfortunately, we did not measure vitamin levels in seminal plasma. Another limitation of this study was the absence of other relevant variables that could potentially influence sperm quality, such as genetic testing (i.e., karyotype and microdeletions of the Y Chromosome). Absence of data regarding the actual intake levels of individual vitamins also weakens the robustness of the conclusions. Future research could combine longitudinal serum monitoring with validated dietary surveys and supplement inventories to delineate the relative contributions of intake, metabolism, and genetic factors to vitamin-associated sperm alterations. Moreover, the baseline information obtained from the questionnaire may induce a recall bias for the results.

## 5 Conclusion

This study provides valuable insights into the joint and non-linear relationship between vitamin levels and sperm quality, holds the potential to guide clinical trials on vitamin supplementation strategies. Certain vitamins, such as B1, B6, B12, and D, appear to have a potential dose-dependent dual effect on sperm quality. While vitamin A might play a detrimental role. Overall, the BKMR models revealed null association between multiple vitamin levels and sperm quality parameters, suggesting that increased of personalized vitamins may be more effective than multivitamins in improving sperm quality. In the future, the role of vitamin supplements in the treatment of male infertility can be further explored.

## Data Availability

The raw data supporting the conclusions of this article will be made available by the authors, without undue reservation.

## References

[1] Levine H Jørgensen N Martino-Andrade A Mendiola J Weksler-Derri D Jolles M. Temporal trends in sperm count: a systematic review and meta-regression analysis of samples collected globally in the 20th and 21st centuries. Hum Reprod Update. (2023) 29:157–76. 10.1093/humupd/dmac035 36377604

[2] Braverman AM Davoudian T Levin IK Bocage A Wodoslawsky S. Depression, anxiety, quality of life, and infertility: a global lens on the last decade of research. Fertil Steril. (2024) 121:379–83. 10.1016/j.fertnstert.2024.01.013 38224730

[3] Huang B Wang Z Kong Y Jin M Ma L. Global, regional and national burden of male infertility in 204 countries and territories between 1990 and 2019: an analysis of global burden of disease study. BMC Public Health. (2023) 23:2195. 10.1186/s12889-023-16793-3 37940907 PMC10631182

[4] Vander Borght M Wyns C. Fertility and infertility: definition and epidemiology. Clin Biochem. (2018) 62:2–10. 10.1016/j.clinbiochem.2018.03.012 29555319

[5] De Jonge CJ Barratt CLR Aitken RJ Anderson RA Baker P Chan DYL. Current global status of male reproductive health. Hum Reprod Open. (2024) 2024:hoae017. 10.1093/hropen/hoae017 38699533 PMC11065475

[6] Crean AJ Afrin S Niranjan H Pulpitel TJ Ahmad G Senior AM. Male reproductive traits are differentially affected by dietary macronutrient balance but unrelated to adiposity. Nat Commun. (2023) 14:2566. 10.1038/s41467-023-38314-x 37142562 PMC10160019

[7] Mandl J Szarka A Bánhegyi G. Vitamin C: update on physiology and pharmacology. Br J Pharmacol. (2009) 157:1097–110. 10.1111/j.1476-5381.2009.00282.x 19508394 PMC2743829

[8] Almujaydil MS. The role of dietary nutrients in male infertility: a review. Life (2023) 13:519. 10.3390/life13020519 36836876 PMC9960932

[9] Banihani SA. Vitamin B12 and semen quality. Biomolecules (2017) 7:42. 10.3390/biom7020042 28598359 PMC5485731

[10] Blomberg Jensen M. Vitamin D and male reproduction. Nat Rev Endocrinol. (2014) 10:175–86. 10.1038/nrendo.2013.262 24419359

[11] Calagna G Catinella V Polito S Schiattarella A De Franciscis P D'Antonio F. Vitamin D and male reproduction: updated evidence based on literature review. Nutrients. (2022) 14:3278. 10.3390/nu14163278 36014783 PMC9412569

[12] Audet I Laforest JP Martineau GP Matte JJ. Effect of vitamin supplements on some aspects of performance, vitamin status, and semen quality in boars. J Anim Sci. (2004) 82:626–33. 10.2527/2004.822626x 14974564

[13] Moeinian N Fathabadi FF Norouzian M Abbaszadeh H-A Nazarian H Afshar A. The effects of vitamin C and vitamin B12 on improving spermatogenesis in mice subjected to long-term scrotal heat stress. Clin Exp Reprod Med. (2024) 51:334–43. 10.5653/cerm.2023.06751 38757278 PMC11617911

[14] Haeri F Nouri M Nezamoleslami S Moradi A Ghiasvand R. Role of dietary antioxidants and vitamins intake in semen quality parameters: a cross-sectional study. Clin Nutr ESPEN. (2022) 48:434–40. 10.1016/j.clnesp.2022.01.005 35331525

[15] Munns CF Shaw N Kiely M Specker BL Thacher TD Ozono K. Global consensus recommendations on prevention and management of nutritional rickets. J Clin Endocrinol Metab. (2016) 101:394–415. 10.1210/jc.2015-2175 26745253 PMC4880117

[16] Venkatesh U Sharma A Ananthan VA Subbiah P Durga R. CSIR Summer Research training team. Micronutrient's deficiency in India: a systematic review and meta-analysis. J Nutr Sci. (2021) 10:e110. 10.1017/jns.2021.102 35059191 PMC8727714

[17] Yao W Liu C Qin D-Y Yuan X-Q Yao Q-Y Li N-J. Associations between phthalate metabolite concentrations in follicular fluid and reproductive outcomes among women undergoing *in vitro* fertilization/intracytoplasmic sperm injection treatment. Environ Health Perspect. (2023) 131:127019. 10.1289/EHP11998 38150316 PMC10752415

[18] Wang C Mbizvo M Festin MP Björndahl L Toskin I other Editorial Board Members of the WHO Laboratory Manual for the Examination and Processing of Human Semen. Evolution of the WHO “Semen” processing manual from the first (1980) to the sixth edition (2021). Fertil Steril (2022) 117:237–45. 10.1016/j.fertnstert.2021.11.037 34996596 PMC8842884

[19] Menkveld R Stander FS Kotze TJ Kruger TF van Zyl JA. The evaluation of morphological characteristics of human spermatozoa according to stricter criteria. Hum Reprod. (1990) 5:586–92. 10.1093/oxfordjournals.humrep.a137150 2394790

[20] Boushaba S Helis Y Lebaal R Beldjebel S Benhamza A Ziti C. The relationship of sperm DNA integrity with serum vitamin levels (folate and cobalamin) and food consumption in infertile men. Clin Exp Reprod Med. (2023) 50:53–62. 10.5653/cerm.2022.05736 36935412 PMC10030204

[21] Qaim M. Benefits of genetically modified crops for the poor: household income, nutrition, and health. N Biotechnol. (2010) 27:552–7. 10.1016/j.nbt.2010.07.009 20643233

[22] Luo X Yin C Shi Y Du C Pan X. Global trends in semen quality of young men: a systematic review and regression analysis. J Assist Reprod Genet. (2023) 40:1807–16. 10.1007/s10815-023-02859-z 37335419 PMC10371917

[23] Li Y Lin H Li Y Cao J. Association between socio-psycho-behavioral factors and male semen quality: systematic review and meta-analyses. Fertil Steril. (2011) 95:116–23. 10.1016/j.fertnstert.2010.06.031 20674912

[24] Mousavi SE Amini H Heydarpour P Amini Chermahini F Godderis L. Air pollution, environmental chemicals, and smoking may trigger vitamin D deficiency: Evidence and potential mechanisms. Environ Int. (2019) 122:67–90. 10.1016/j.envint.2018.11.052 30509511

[25] Gibson A Woodside JV Young IS Sharpe PC Mercer C Patterson CC. J, Whitehead AS, Evans A. Alcohol increases homocysteine and reduces B vitamin concentration in healthy male volunteers–a randomized, crossover intervention study. QJM. (2008) 101:881–7. 10.1093/qjmed/hcn112 18790817 PMC2572692

[26] Trollfors B. Ethnicity, gender and seasonal variations all play a role in vitamin D deficiency. Acta Paediatr. (2022) 111:1596–602. 10.1111/apa.16372 35472253

[27] Fu Y Zhu Z Huang Z He R Zhang Y Li Y. Association between vitamin b and obesity in middle-aged and older chinese adults. Nutrients. (2023) 15:483. 10.3390/nu15030483 36771189 PMC9921635

[28] Bobb JF Claus Henn B Valeri L Coull BA. Statistical software for analyzing the health effects of multiple concurrent exposures via Bayesian kernel machine regression. Environ Health. (2018) 17:67. 10.1186/s12940-018-0413-y 30126431 PMC6102907

[29] Whitfield KC Bourassa MW Adamolekun B Bergeron G Bettendorff L Brown KH. Thiamine deficiency disorders: diagnosis, prevalence, and a roadmap for global control programs. Ann N Y Acad Sci. (2018) 1430:3–43. 10.1111/nyas.13919 30151974 PMC6392124

[30] Chan Y-M Bailey R O'Connor DL. Folate. Adv Nutr. (2013) 4:123–5. 10.3945/an.112.003392 23319130 PMC3648733

[31] Diab L Krebs NF. Vitamin Excess and Deficiency. Pediatr Rev. (2018) 39:161–79. 10.1542/pir.2016-0068 29610425

[32] Yang C Chen J Liu Z Yun C Piao J Yang X. Prevalence and influence factors of vitamin A deficiency of Chinese pregnant women. Nutr J. (2016) 15:12. 10.1186/s12937-016-0131-7 26818747 PMC4729160

[33] Braadland PR Bergquist A Kummen M Bossen L Engesæter LK Reims HM. Clinical and biochemical impact of vitamin B6 deficiency in primary sclerosing cholangitis before and after liver transplantation. J Hepatol. (2023) 79:955–66. 10.1016/j.jhep.2023.05.038 37328069

[34] Gariballa S. Poor vitamin C status is associated with increased depression symptoms following acute illness in older people. Int J Vitam Nutr Res. (2014) 84:12–7. 10.1024/0300-9831/a000188 25835231

[35] Sapiejka E Krzyżanowska-Jankowska P Wenska-Chyży E Szczepanik M Walkowiak D Cofta S. Vitamin E status and its determinants in patients with cystic fibrosis. Adv Med Sci. (2018) 63:341–6. 10.1016/j.advms.2018.04.001 30081288

[36] McNulty H Pentieva K Ward M. Causes and clinical sequelae of riboflavin deficiency. Annu Rev Nutr. (2023) 43:101–22. 10.1146/annurev-nutr-061121-084407 37603429

[37] WHO. Micronutrients. Available online at: https://www.who.int/health-topics/micronutrients (accessed July 30, 2024).

[38] Chen Y Liu D Zeng L Xu H Jiang H Yang R. Effect of serum 25-hydroxyvitamin D levels on sperm quality and assisted reproductive technology outcomes for men of infertile Chinese couples. Andrology. (2020) 8:1277–86. 10.1111/andr.12818 32412142

[39] Abbasihormozi S Kouhkan A Alizadeh AR Shahverdi AH Nasr-Esfahani MH Sadighi Gilani MA. Association of vitamin D status with semen quality and reproductive hormones in Iranian subfertile men. Andrology. (2017) 5:113–8. 10.1111/andr.12280 27792863

[40] Hajianfar H Karimi E Mollaghasemi N Rezaei S Arab A. Is there a relationship between serum vitamin D and semen parameters? A cross-sectional sample of the Iranian infertile men. Basic Clin Androl. (2021) 31:29. 10.1186/s12610-021-00147-3 34852757 PMC8638431

[41] Blaseg E Von Wald T Hansen KA. Vitamin D levels and human sperm DNA fragmentation: a prospective, cohort study. Basic Clin Androl. (2022) 32:14. 10.1186/s12610-022-00166-8 36096748 PMC9469602

[42] Shahreza FD Hajian M Gharagozloo P Drevet JR Nasr-Esfahani MH. Impact of vitamin D deficiency on mouse sperm structure and function. Andrology. (2020) 8:1442–55. 10.1111/andr.12820 32421931

[43] Liang Y Lu J Yi W Cai M Shi W Li B. 1α,25-dihydroxyvitamin D3 supplementation alleviates perfluorooctanesulfonate acid-induced reproductive injury in male mice: Modulation of Nrf2 mediated oxidative stress response. Environ Toxicol. (2023) 38:322–31. 10.1002/tox.23685 36321694

[44] Pouriayevali F Tavalaee M Taktaz-Hafshejani T Dattilio M Nasr-Esfahani MH. Overlapping sperm damages from vitamin B or D deficiency in mice: insights into the role of clinical supplementations. Andrologia. (2022) 54:e14592. 10.1111/and.14592 36123798

[45] Homayouni-Meymandi M Sotoodehnejadnematalahi F Nasr-Esfahani MH. Relationship between serum vitamin d in male, sperm function and clinical outcomes in infertile men candidate for ICSI: a cohort study. Int J Fertil Steril. (2022) 16:115–21. 10.22074/IJFS.2021.522049 35639649 PMC9108299

[46] Dabagh M Jahangiri N Taheri Madah A Rostami S Amidi F Khodarahmian M. Association of dietary total antioxidant capacity, alternative healthy eating index, and dietary inflammatory index with semen quality in men seeking infertility treatment. Front Nutr. (2023) 10:1284379. 10.3389/fnut.2023.1284379 37885439 PMC10598851

[47] Fleming JC Tartaglini E Kawatsuji R Yao D Fujiwara Y Bednarski JJ. Male infertility and thiamine-dependent erythroid hypoplasia in mice lacking thiamine transporter Slc19a2. Mol Genet Metab. (2003) 80:234–41. 10.1016/S1096-7192(03)00141-0 14567973

[48] Ghyasvand T Goodarzi MT Amiri I Karimi J Ghorbani M. Serum levels of lycopene, beta-carotene, and retinol and their correlation with sperm DNA damage in normospermic and infertile men. Int J Reprod Biomed. (2015) 13:787–92. 10.29252/ijrm.13.12.787 27141539 PMC4827510

[49] Omu AE Fatinikun T Mannazhath N Abraham S. Significance of simultaneous determination of serum and seminal plasma alpha-tocopherol and retinol in infertile men by high-performance liquid chromatography. Andrologia. (1999) 31:347–54. 10.1046/j.1439-0272.1999.00296.x 10643509

[50] Schisterman EF Sjaarda LA Clemons T Carrell DT Perkins NJ Johnstone E. Effect of folic acid and zinc supplementation in men on semen quality and live birth among couples undergoing infertility treatment: a randomized clinical trial. JAMA. (2020) 323:35–48. 10.1001/jama.2019.18714 31910279 PMC6990807

[51] Chihara M Otsuka S Ichii O Kon Y. Vitamin A deprivation affects the progression of the spermatogenic wave and initial formation of the blood-testis barrier, resulting in irreversible testicular degeneration in mice. J Reprod Dev. (2013) 59:525–35. 10.1262/jrd.2013-058 23934320 PMC3934156

[52] Banihani SA A. Systematic review evaluating the effect of vitamin B6 on semen quality. Urol J. (2017) 15:1–5. 10.22037/uj.v0i0.3808 29290084

[53] Zhou X Shi H Zhu S Wang H Sun S. Effects of vitamin E and vitamin C on male infertility: a meta-analysis. Int Urol Nephrol. (2022) 54:1793–805. 10.1007/s11255-022-03237-x 35604582

[54] Gangolf M Czerniecki J Radermecker M Detry O Nisolle M Jouan C. Thiamine status in humans and content of phosphorylated thiamine derivatives in biopsies and cultured cells. PLoS ONE. (2010) 5:e13616. 10.1371/journal.pone.0013616 21049048 PMC2963613

[55] Sadeghi N Boissonneault G Tavalaee M Nasr-Esfahani MH. Oxidative vs. reductive stress: a delicate balance for sperm integrity. Syst Biol Reprod Med. (2023) 69:20–31. 10.1080/19396368.2022.2119181 36215401

[56] Froese DS Fowler B Baumgartner MR. Vitamin B12, folate, and the methionine remethylation cycle-biochemistry, pathways, and regulation. J Inherit Metab Dis. (2019) 42:673–85. 10.1002/jimd.12009 30693532

[57] Glier MB Green TJ Devlin AM. Methyl nutrients, DNA methylation, and cardiovascular disease. Mol Nutr Food Res. (2014) 58:172–82. 10.1002/mnfr.201200636 23661599

[58] Blomberg Jensen M Nielsen JE Jørgensen A Rajpert-De Meyts E Kristensen DM Jørgensen N. Vitamin D receptor and vitamin D metabolizing enzymes are expressed in the human male reproductive tract. Hum Reprod. (2010) 25:1303–11. 10.1093/humrep/deq024 20172873

[59] Wang L Lu H Wang S Liu H Guo M Bai H. Vitamin D Receptor affects male mouse fertility via regulation of lipid metabolism and testosterone biosynthesis in testis. Gene. (2022) 834:146589. 10.1016/j.gene.2022.146589 35598688

[60] Zhou Y Wang Y. Action and interaction between retinoic acid signaling and blood-testis barrier function in the spermatogenesis cycle. Cells. (2022) 11:352. 10.3390/cells11030352 35159162 PMC8834282

[61] Bdeir R Aljabali SM Banihani SA. Role of pyridoxine and oxidative stress in asthenozoospermia. Heliyon. (2024) 10:e34799. 10.1016/j.heliyon.2024.e34799 39148988 PMC11325350

[62] Kobori Y Ota S Sato R Yagi H Soh S Arai G. Antioxidant cosupplementation therapy with vitamin C, vitamin E, and coenzyme Q10 in patients with oligoasthenozoospermia. Arch Ital Urol Androl. (2014) 86:1–4. 10.4081/aiua.2014.1.1 24704922

[63] Yokota S Sekine N Wakayama T Oshio S. Impact of chronic vitamin A excess on sperm morphogenesis in mice. Andrology. (2021) 9:1579–92. 10.1111/andr.13013 33818007

[64] Alshahrani F Aljohani N. Vitamin D: deficiency, sufficiency and toxicity. Nutrients. (2013) 5:3605–16. 10.3390/nu5093605 24067388 PMC3798924

[65] Yang Y Yang L Han X Wu K Mei G Wu B. The regulation role of calcium channels in mammalian sperm function: a narrative review with a focus on humans and mice. PeerJ. (2024) 12:e18429. 10.7717/peerj.18429 39469589 PMC11514763

[66] Jueraitetibaike K Ding Z Wang D-D Peng L-P Jing J Chen L. The effect of vitamin D on sperm motility and the underlying mechanism. Asian J Androl. (2019) 21:400–7. 10.4103/aja.aja_105_18 30618415 PMC6628736

