# Supplementary material for: Joint and individual associations between multiple vitamins and sperm quality in adult men
Source: Front Nutr. 2025 Mar 28;12:1534309. doi: 10.3389/fnut.2025.1534309 (PMC11985434; doi:10.3389/fnut.2025.1534309)
Supplement: Supplementary file 1 [file Table_1.docx]

**Supplementary Materials**

**Joint and individual associations between multiple vitamins and sperm quality in adult men**

Wen Yao, Juan Zhang, Weihong Yan, Di Jie, Ping Tuo, Jie Liu, Xiaoling Zhao, Yiwen Xiong, Yang Li, and Tiejun Pan

**Tables of Contents**

**Table S1.** Basic characteristics of males included in this study.

Table S2. Crude associations between vitamin concentrations in serum and sperm quality.

Table S3. Associations between vitamin concentrations in serum and sperm quality among normospermic males.

**Table S4.** Estimate effects (95% credible intervals) of the mixtures of vitamins on sperm parameters by BKMR models when all the vitamin concentrations at particular percentiles were compared to all the vitamins at their 50th percentile.

**Table S5.** Posterior inclusion probabilities (PIPs) for group inclusion and conditional inclusion of the parameters of sperm quality.

**Figure legends**

**Figure S1.** Flowchart of study population.

**Figure S2.** Directed acyclic graph (DAG) of potential confounder.

**Figure S3.** Correlation coefficients of Spearman correlation analysis for nine vitamins in serum.

**Figure S4.** The nonlinear associations between vitamins (B1, B2, B6, B9, B12, C, A, D, and E) and sperm parameters (total sperm number, sperm concentration, progressive motile sperm, morphologically normal, and DFI).

**Figure** S5. Univariate exposure-response relationships and 95% credible intervals between concentrations of individual vitamins and (A) total sperm number, (B) sperm concentration, (C) progressive motile sperm, (D) morphologically normal rate, (E) DFI when fixing other phthalate metabolites at their median values.

| Table S1. Basic characteristics of males included in this study. | | | |
| --- | --- | --- | --- |
| Basic characteristics | Males in this study | All males | P value |
|  | Mean±SD or N (%) | Mean±SD or N (%) |  |
| Age (years)^a^ | 30.5±3.3 | 30.9±3.7 | 0.33 |
| Height(m) | 1.7±0.1 | 1.7±0.1 | 0.96 |
| Weight(kg) | 72.0±8.7 | 72.3±9.5 | 0.75 |
| BMI (kg/m^2^)^a^ | 23.9±2.5 | 24.0±2.6 | 0.72 |
| Duration of abstinence (day) | 4.7±1.8 | 4.7±1.8 | 0.71 |
| Ethnicity |  |  |  |
| Han | 144 (92.3%) | 440 (93.6%) | 1.00 |
| Other Chinese minorities^b^ | 12 (7.7%) | 30 (6.4%) |  |
| Education level |  |  |  |
| High school and below | 23 (14.7%) | 18 (5.7%) | 0.43 |
| College and above | 133 (85.3%) | 296 (94.3%) |  |
| Household income (Yuan/month)^a^ |  |  |  |
| ≤5000 (reference) | 11 (7.1%) | 38 (12.1%) | 0.24 |
| 5001–10000 | 53 (34.0%) | 99 (31.5%) |  |
| ≥10001 | 92 (59.0%) | 177 (56.4%) |  |
| Smoking status^a^ |  |  |  |
| Never(reference) | 79 (50.6%) | 156 (49.7%) | 0.81 |
| Current | 59 (37.8%) | 115 (36.6%) |  |
| Quit | 18 (11.5%) | 43 (13.7%) |  |
| Drinking status^a^ |  |  |  |
| Never (reference) | 42 (26.9%) | 85 (27.1%) | 0.97 |
| Current | 102 (65.4%) | 203 (64.6%) |  |
| Quit | 12 (7.7%) | 26 (8.3%) |  |
| Note: 1 male was missing for height in calculating BMI so we used the median of height to impute it.  ^a^Covariates included in the multivariable models.  ^b^Other Chinese minorities included Dong, Hui, GeLao, LiSu, Mongolian, TuJia, and Zhuang nationalities.  Abbreviations: BMI, body mass index; SD, standard deviation. | | | |

| Table S2. Crude associations between vitamin concentrations in serum and sperm quality. | | | | | |
| --- | --- | --- | --- | --- | --- |
| Vitamins | Total sperm number  (n=156) | Sperm concentration  (n=156) | Progressive motile sperm (n=156) | Morphologically normal rate (n=147) | DFI  (n=140) |
|  | β (95% CI) | β (95% CI) | β (95% CI) | β (95% CI) | β (95% CI) |
| Vitamin B1 (nmol/L) |  |  |  |  |  |
| Q1(<64.0) | ref | ref | ref | ref | ref |
| Q2(64.0–76.0) | 1.8 (–195.2, 198.9) | –10.1 (–50.7, 30.6) | –2.2 (–8.1, 3.7) | 0.0 (–0.5, 0.5) | 0.3 (–1.9, 2.5) |
| Q3(>76.0) | 228.8 (32.6, 424.9) | 40.7 (0.2, 81.2) | –3.8 (–9.7, 2.1) | 0.1 (–0.5, 0.6) | 2.1 (–0.1, 4.2) |
| P for trend | 0.02 | 0.05 | 0.20 | 0.84 | 0.06 |
| Vitamin B2 (μg/L) |  |  |  |  |  |
| Q1(<225.6) | ref | ref | ref | ref | ref |
| Q2(225.6–278.0) | –29.3 (–230.8, 172.2) | 5.8 (–35.8, 47.4) | 3.3 (–2.6, 9.2) | –0.2 (–0.8, 0.3) | –1.1 (–3.3, 1.1) |
| Q3(>278.0) | –24.2 (–223.8, 175.4) | 2.5 (–38.7, 43.7) | 1.4 (–4.4, 7.3) | –0.4 (–0.9, 0.1) | –0.9 (–3.1, 1.3) |
| P for trend | 0.81 | 0.91 | 0.63 | 0.14 | 0.41 |
| Vitamin B6 (μmol/L) |  |  |  |  |  |
| Q1(<16.5) | ref | ref | ref | ref | ref |
| Q2(16.5–21.7) | –67.4 (–270.7, 135.9) | –11.7 (–53.7, 30.2) | 4.8 (–1.2, 10.7) | 0.1 (–0.4, 0.7) | 0.3 (–1.9, 2.5) |
| Q3(>21.7) | –6.0 (–206.6, 194.6) | –16.8 (–58.2, 24.6) | 3.9 (–2.0, 9.7) | 0.1 (–0.5, 0.6) | –0.4 (–2.6, 1.8) |
| P for trend | 0.97 | 0.43 | 0.20 | 0.78 | 0.73 |
| Vitamin B9 (nmol/L) |  |  |  |  |  |
| Q1(<15.1) | ref | ref | ref | ref | ref |
| Q2(15.1–19.2) | –26.5 (–226.6, 173.6) | 4.5 (–36.8, 45.7) | 3.5 (–2.3, 9.4) | 0.0 (–0.5, 0.5) | –0.4 (–2.6, 1.8) |
| Q3(>19.2) | –90.8 (–291.8, 110.2) | –15.2 (–56.7, 26.3) | 2.0 (–3.9, 7.9) | –0.2 (–0.8, 0.3) | –0.6 (–2.8, 1.6) |
| P for trend | 0.37 | 0.47 | 0.5 | 0.39 | 0.60 |
| Vitamin B12 (pg/mL) |  |  |  |  |  |
| Q1(<311.6) | ref | ref | ref | ref | ref |
| Q2(311.6–418.0) | –70.8 (–272.0, 130.5) | –36.0 (–77.1, 5.2) | 1.2 (–4.7, 7.1) | –0.3 (–0.8, 0.2) | –0.7 (–2.8, 1.4) |
| Q3(>418.0) | –20.4 (–219.7, 178.9) | –9.9 (–50.7, 30.8) | –0.1 (–6.0, 5.8) | –0.1 (–0.6, 0.4) | 3.0 (0.9, 5.1) |
| P for trend | 0.84 | 0.64 | 0.97 | 0.71 | <0.01 |
| Vitamin C (μmol/L) |  |  |  |  |  |
| Q1(<36.0) | ref | ref | ref | ref | ref |
| Q2(36.0–43.0) | –17.8 (–221.1, 185.5) | –12.4 (–54.4, 29.5) | –1.9 (–7.8, 4.1) | –0.4 (–0.9, 0.1) | 0.5 (–1.7, 2.7) |
| Q3(>43.0) | 36.8 (–168.2, 241.8) | –11.0 (–53.3, 31.3) | –0.6 (–6.6, 5.5) | 0.1 (–0.5, 0.6) | –1.0 (–3.2, 1.2) |
| P for trend | 0.71 | 0.62 | 0.88 | 0.82 | 0.36 |
| Vitamin A (μmol/L) |  |  |  |  |  |
| Q1(<0.5) | ref | ref | ref | ref | ref |
| Q2(0.5–0.7) | –40.1 (–240.5, 160.4) | –1.2 (–42.5, 40.2) | 1.7 (–4.1, 7.4) | –0.3 (–0.8, 0.2) | –0.9 (–3.1, 1.3) |
| Q3(>0.7) | –43.7 (–245.1, 157.7) | –5.1 (–46.7, 36.5) | –6.0 (–11.8, –0.2) | –0.1 (–0.6, 0.4) | 0.6 (–1.6, 2.8) |
| P for trend | 0.67 | 0.81 | 0.04 | 0.74 | 0.60 |
| 1,25-(OH)2-D3 (nmol/L) |  |  |  |  |  |
| Q1(<28.6) | ref | ref | ref | ref | ref |
| Q2(28.6–37.0) | 0.7 (–204.8, 206.2) | –13.4 (–56.0, 29.3) | –2.3 (–8.4, 3.8) | 0.2 (–0.4, 0.7) | 0.8 (–1.4, 3.1) |
| Q3(>37.0) | –130.2 (–324.1, 63.7) | –15.9 (–56.1, 24.3) | 0.7 (–5.0, 6.4) | 0.5 (0.0, 1.0) | 1.0 (–1.1, 3.2) |
| P for trend | 0.18 | 0.44 | 0.79 | 0.05 | 0.35 |
| Vitamin E (μg/mL) |  |  |  |  |  |
| Q1(<12.0) | ref | ref | ref | ref | ref |
| Q2(12.0–13.0) | 81.5 (–124.5, 287.5) | 11.1 (–31.2, 53.5) | 0.0 (–6.1, 6.0) | 0.3 (–0.2, 0.9) | 1.3 (–0.9, 3.6) |
| Q3(>13.0) | 63.1 (–140.5, 266.6) | 29.5 (–12.3, 71.3) | 1.7 (–4.3, 7.7) | 0.3 (–0.2, 0.9) | 1.6 (–0.6, 3.8) |
| P for trend | 0.57 | 0.16 | 0.56 | 0.23 | 0.17 |
| Note: Linear regression models without covariates. *P* for trend was derived by entering the quartiles of exposure as ordinal categorical variables (1–3) in the models. The level of 1,25(OH)_2_D3 in serum represents vitamin D status in human.  Q1, Q2 and Q3 refer to the tertile (1-3) concentrations of the 156 volunteers in this study.  Abbreviations: BMI, body mass index; DFI, DNA fragmetation index. | | | | | |

| Table S3. Associations between vitamin concentrations in serum and sperm quality among normospermic males. | | | | | |
| --- | --- | --- | --- | --- | --- |
| Vitamins | Total sperm number  (n=104) | Sperm concentration  (n=104) | Progressive motile sperm (n=104) | Morphologically normal (n=101) | DFI  (n=97) |
|  | β (95% CI) | β (95% CI) | β (95% CI) | β (95% CI) | β (95% CI) |
| Vitamin B1 (nmol/L) |  |  |  |  |  |
| Q1(<64.0) | ref | ref | ref | ref | ref |
| Q2(64.0–76.0) | 56.4 (–147.1, 259.8) | –10.5 (–60.0, 39.0) | 2.2 (–2.6, 7.0) | 0.00 (–0.7, 0.7) | –1.2 (–3.4, 1.0) |
| Q3(>76.0) | 206.6 (5.5, 407.7) | 37.0 (–12.0, 85.9) | –3.6 (–8.4, 1.1) | –0.2 (–0.9, 0.5) | 1.3 (–0.8, 3.5) |
| P for trend | 0.04 | 0.14 | 0.14 | 0.57 | 0.22 |
| Vitamin B2 (μg/L) |  |  |  |  |  |
| Q1(<225.6) | ref | ref | ref | ref | ref |
| Q2(225.6–278.0) | –33.2 (–248.5, 182.2) | 8.9 (–43.4, 61.2) | –1.6 (–6.7, 3.5) | –0.2 (–0.9, 0.5) | 0.6 (–1.7, 3.0) |
| Q3(>278.0) | –65.9 (–274.1, 142.3) | –8.9 (–59.4, 41.6) | 1.5 (–3.4, 6.4) | –0.5 (–1.2, 0.2) | 0.8 (–1.5, 3.0) |
| P for trend | 0.53 | 0.71 | 0.52 | 0.14 | 0.51 |
| Vitamin B6 (μmol/L) |  |  |  |  |  |
| Q1(<16.5) | ref | ref | ref | ref | ref |
| Q2(16.5–21.7) | 20.4 (–195.0, 235.7) | 0.1 (–52.1, 52.4) | 1.3 (–3.8, 6.4) | –0.3 (–1.0, 0.4) | –0.2 (–2.5, 2.1) |
| Q3(>21.7) | 35.3 (–168.7, 239.4) | –9.9 (–59.5, 39.6) | 1.3 (–3.6, 6.1) | 0.3 (–0.4, 1.0) | –0.6 (–2.9, 1.6) |
| P for trend | 0.73 | 0.68 | 0.61 | 0.34 | 0.56 |
| Vitamin B9 (nmol/L) |  |  |  |  |  |
| Q1(<15.1) | ref | ref | ref | ref | ref |
| Q2(15.1–19.2) | 97.5 (–113.3, 308.4) | 21.2 (–29.8, 72.2) | 3.6 (–1.4, 8.6) | –0.2 (–0.8, 0.5) | –0.3 (–2.6, 2.0) |
| Q3(>19.2) | –39.0 (–252.3, 174.3) | –19.3 (–70.9, 32.3) | –0.4 (–5.4, 4.6) | –0.3 (–1.0, 0.4) | 0.2 (–2.1, 2.5) |
| P for trend | 0.70 | 0.44 | 0.85 | 0.43 | 0.87 |
| Vitamin B12 (pg/mL) |  |  |  |  |  |
| Q1(<311.6) | ref | ref | ref | ref | ref |
| Q2(311.6–418.0) | –108.4 (–320.2, 103.5) | –58.7 (–108.9, –8.4) | 0.0 (–5.0, 5.1) | –0.2 (–0.9, 0.6) | 0.1 (–2.1, 2.3) |
| Q3(>418.0) | –53.9 (–263.6, 155.9) | –34.5 (–84.3, 15.3) | 0.8 (–4.3, 5.8) | –0.2 (–0.9, 0.5) | 3.2 (1.0, 5.4) |
| P for trend | 0.60 | 0.17 | 0.77 | 0.54 | <0.01 |
| Vitamin C (μmol/L) |  |  |  |  |  |
| Q1(<36.0) | ref | ref | ref | ref | ref |
| Q2(36.0–43.0) | –21.7 (–236.6, 193.2) | –8.9 (–61.6, 43.9) | –2.9 (–8.0, 2.3) | 0.0 (–0.7, 0.7) | 1.3 (–1.0, 3.6) |
| Q3(>43.0) | 121.6 (–89.1, 332.2) | –6.7 (–58.4, 45.0) | –1.4 (–6.4, 3.7) | 0.5 (–0.2, 1.2) | 0.6 (–1.8, 2.9) |
| P for trend | 0.22 | 0.81 | 0.65 | 0.13 | 0.70 |
| Vitamin A (μmol/L) |  |  |  |  |  |
| Q1(<0.5) | ref | ref | ref | ref | ref |
| Q2(0.5–0.7) | –8.4 (–221.0, 204.2) | 8.7 (–42.9, 60.2) | 1.3 (–3.7, 6.4) | –0.7 (–1.4, 0.0) | –0.7 (–2.9, 1.6) |
| Q3(>0.7) | 5.1 (–202.9, 213.1) | 9.1 (–41.4, 59.6) | 0.0 (–4.9, 5.0) | –0.2 (–0.8, 0.5) | –0.8 (–3.0, 1.5) |
| P for trend | 0.96 | 0.72 | 0.99 | 0.63 | 0.49 |
| 1,25-(OH)2-D3 (nmol/L) |  |  |  |  |  |
| Q1(<28.6) | ref | ref | ref | ref | ref |
| Q2(28.6–37.0) | –103.6 (–312.4, 105.2) | –33.5 (–84.1, 17.1) | –1.1 (–6.1, 3.9) | 0.1 (–0.6, 0.8) | –0.7 (–3.0, 1.5) |
| Q3(>37.0) | –111.9 (–315.7, 91.8) | –21.3 (–70.6, 28.1) | 1.4 (–3.5, 6.2) | 0.4 (–0.2, 1.1) | 0.7 (–1.5, 2.9) |
| P for trend | 0.28 | 0.41 | 0.56 | 0.18 | 0.50 |
| Vitamin E (μg/mL) |  |  |  |  |  |
| Q1(<12.0) | ref | ref | ref | ref | ref |
| Q2(12.0–13.0) | 131.5 (–90.1, 353.1) | 30.3 (–23.5, 84.2) | 2.9 (–2.4, 8.2) | 0.3 (–0.5, 1.0) | 0.6 (–1.8, 2.9) |
| Q3(>13.0) | 168.7 (–34.7, 372.0) | 39.0 (–10.4, 88.5) | 0.3 (–4.6, 5.2) | 0.5 (–0.1, 1.2) | 2.3 (0.2, 4.5) |
| P for trend | 0.11 | 0.13 | 0.99 | 0.10 | 0.03 |
| Note: Linear regression models were adjusted for age, BMI, household income, smoke status, and drinking status. *P* for trend was derived by entering the quartiles of exposure as ordinal categorical variables (1–3) in the models. The level of 1,25(OH)_2_D3 in serum represents vitamin D status in human.  Q1, Q2 and Q3 refer to the tertile (1-3) concentrations of the 156 volunteers in this study.  Abbreviations: BMI, body mass index; DFI, DNA fragmetation index. | | | | | |

| **Table S4.** Estimate effects (95% credible intervals) of the mixtures of vitamins on sperm parameters by BKMR models when all the vitamin concentrations at particular percentiles were compared to all the vitamins at their 50th percentile. | | | | | |
| --- | --- | --- | --- | --- | --- |
| Quantile of the mixtures | Total sperm number | Sperm concentration | Progressive motile sperm | Morphologically normal rate | DFI |
|  | β (95% credible intervals) | β (95% credible intervals) | β (95% credible intervals) | β (95% credible intervals) | β (95% credible intervals) |
| 25 | –16.37 (–152.36, 119.63) | –3.66 (–32.38, 25.07) | –1.76 (–5.74, 2.22) | –0.27 (–0.63, 0.10) | –0.06 (–0.44, 0.32) |
| 30 | 1.84 (–95.24, 98.93) | 3.37 (–16.60, 23.34) | –1.04 (–3.82, 1.74) | –0.12 (–0.39, 0.15) | –0.03 (–0.28, 0.22) |
| 35 | –3.00 (–67.95, 61.94) | 2.24 (–11.41, 15.89) | –0.69 (–2.63, 1.25) | –0.06 (–0.25, 0.13) | –0.02 (–0.19, 0.16) |
| 40 | 1.76 (–43.55, 47.06) | 1.65 (–7.89, 11.19) | –0.41 (–1.76, 0.94) | –0.03 (–0.16, 0.10) | –0.01 (–0.14, 0.11) |
| 45 | 3.40 (–21.61, 28.41) | 1.22 (–4.00, 6.44) | –0.34 (–1.07, 0.39) | –0.02 (–0.10, 0.05) | 0.00 (–0.07, 0.06) |
| 50 | 0.00 (0.00, 0.00) | 0.00 (0.00, 0.00) | 0.00 (0.00, 0.00) | 0.00 (0.00, 0.00) | 0.00 (0.00, 0.00) |
| 55 | –7.35 (–40.49, 25.79) | –2.09 (–9.28, 5.11) | 0.58 (–0.51, 1.67) | –0.01 (–0.09, 0.08) | 0.01 (–0.07, 0.08) |
| 60 | –8.34 (–64.18, 47.50) | –2.61 (–14.49, 9.27) | 0.64 (–1.09, 2.37) | 0.02 (–0.12, 0.17) | 0.01 (–0.12, 0.15) |
| 65 | 0.93 (–100.25, 102.11) | 3.48 (–18.35, 25.31) | 1.42 (–1.68, 4.52) | 0.13 (–0.15, 0.40) | 0.04 (–0.27, 0.36) |
| 75 | –0.11 (–118.10, 117.88) | 2.61 (–22.73, 27.95) | 1.45 (–2.18, 5.07) | 0.13 (–0.20, 0.45) | 0.05 (–0.31, 0.41) |
| Note: BKMR models were adjusted for age, BMI, household income, smoke status, and drinking status. The level of 1,25–(OH)_2_–D3 in serum represents vitamin D status in human.    Abbreviations: BMI, body mass index; BKMR, Bayesian kernel machine regression; DFI, DNA fragmetation index. | | | | | |

| **Table S5.** Posterior inclusion probabilities (PIPs) for group inclusion and conditional inclusion of the parameters of sperm quality. | | | | | | | | | | | |
| --- | --- | --- | --- | --- | --- | --- | --- | --- | --- | --- | --- |
| Vitamins | group | Total sperm number | | Sperm concentration | | Progressive motile sperm | | Morphologically normal rate | | DFI | |
|  |  | groupPIP | condPIP | groupPIP | condPIP | groupPIP | condPIP | groupPIP | condPIP | groupPIP | condPIP |
| Vitamin B1 | 1 | 0.38 | 0.39 | 0.37 | 0.26 | 0.39 | 0.09 | 0.27 | 0.11 | 0.70 | 0.17 |
| Vitamin B2 | 1 | 0.38 | 0.10 | 0.37 | 0.12 | 0.39 | 0.19 | 0.27 | 0.12 | 0.70 | 0.15 |
| Vitamin B6 | 1 | 0.38 | 0.11 | 0.37 | 0.17 | 0.39 | 0.41 | 0.27 | 0.32 | 0.70 | 0.17 |
| Vitamin B9 | 1 | 0.38 | 0.16 | 0.37 | 0.19 | 0.39 | 0.11 | 0.27 | 0.10 | 0.70 | 0.16 |
| Vitamin B12 | 1 | 0.38 | 0.11 | 0.37 | 0.11 | 0.39 | 0.08 | 0.27 | 0.20 | 0.70 | 0.18 |
| Vitamin C | 1 | 0.38 | 0.12 | 0.37 | 0.13 | 0.39 | 0.12 | 0.27 | 0.15 | 0.70 | 0.16 |
| Vitamin A | 2 | 0.53 | 0.15 | 0.51 | 0.35 | 0.54 | 0.80 | 0.59 | 0.53 | 0.70 | 0.34 |
| 1,25-(OH)_2_-D_3_ | 2 | 0.53 | 0.67 | 0.51 | 0.32 | 0.54 | 0.10 | 0.59 | 0.28 | 0.70 | 0.33 |
| Vitamin E | 2 | 0.53 | 0.18 | 0.51 | 0.33 | 0.54 | 0.11 | 0.59 | 0.18 | 0.70 | 0.33 |
| Note: BKMR models were adjusted for age, BMI, household income, smoke status, and drinking status. The level of 1,25–(OH)_2_–D3 in serum represents vitamin D status in human.    Abbreviations: BMI, body mass index; BKMR, Bayesian kernel machine regression; DFI, DNA fragmetation index. | | | | | | | | | | | |


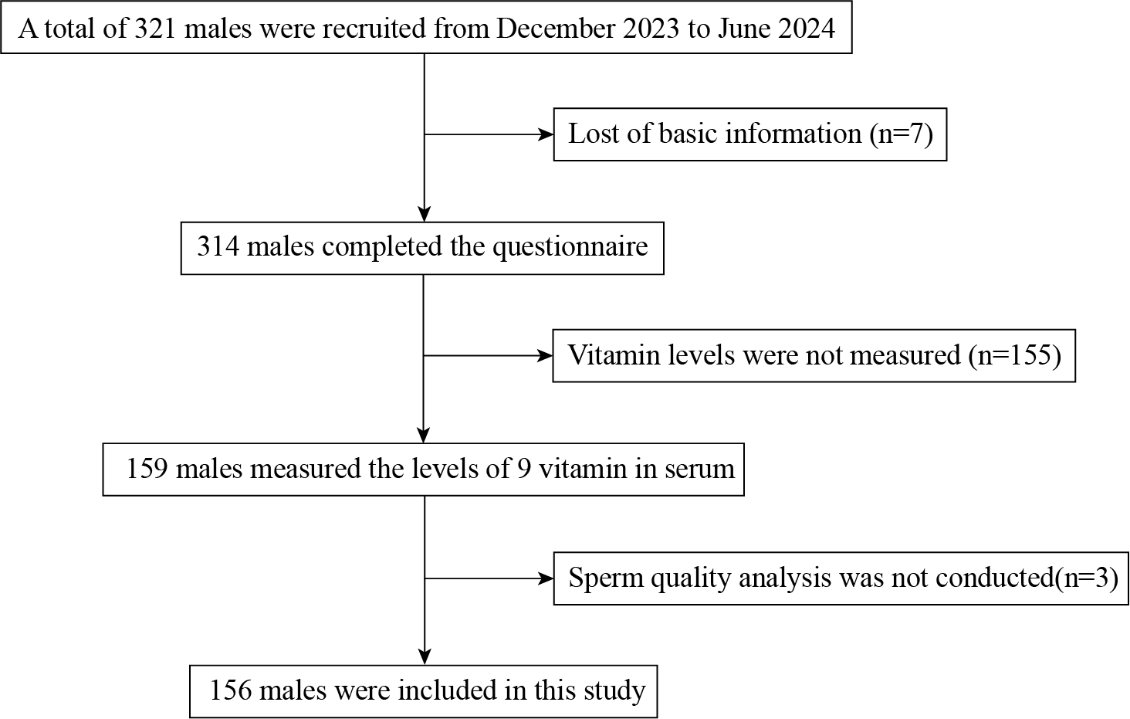


**Figure S1.** Flowchart of study population.


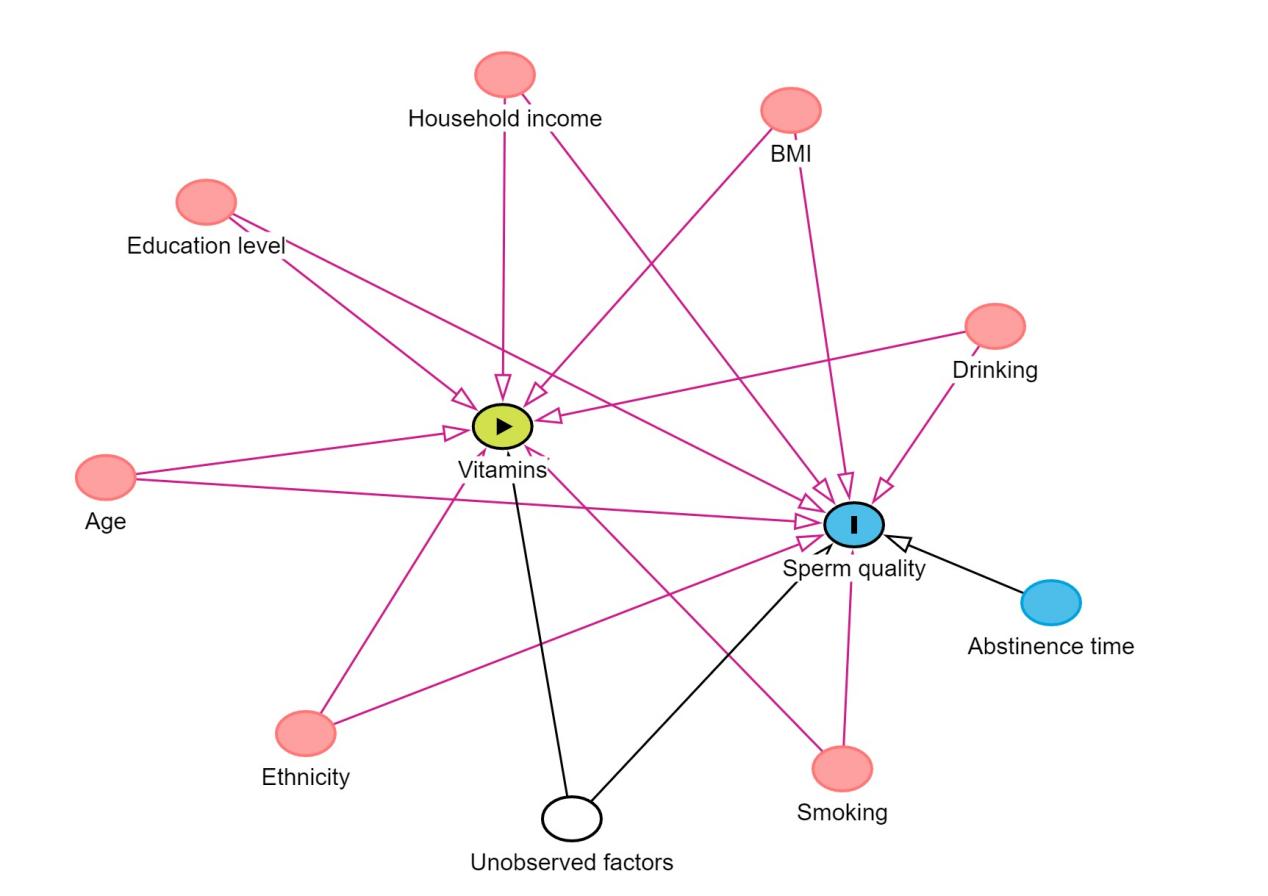


**Figure S2.** Directed acyclic graph (DAG) of potential confounders.


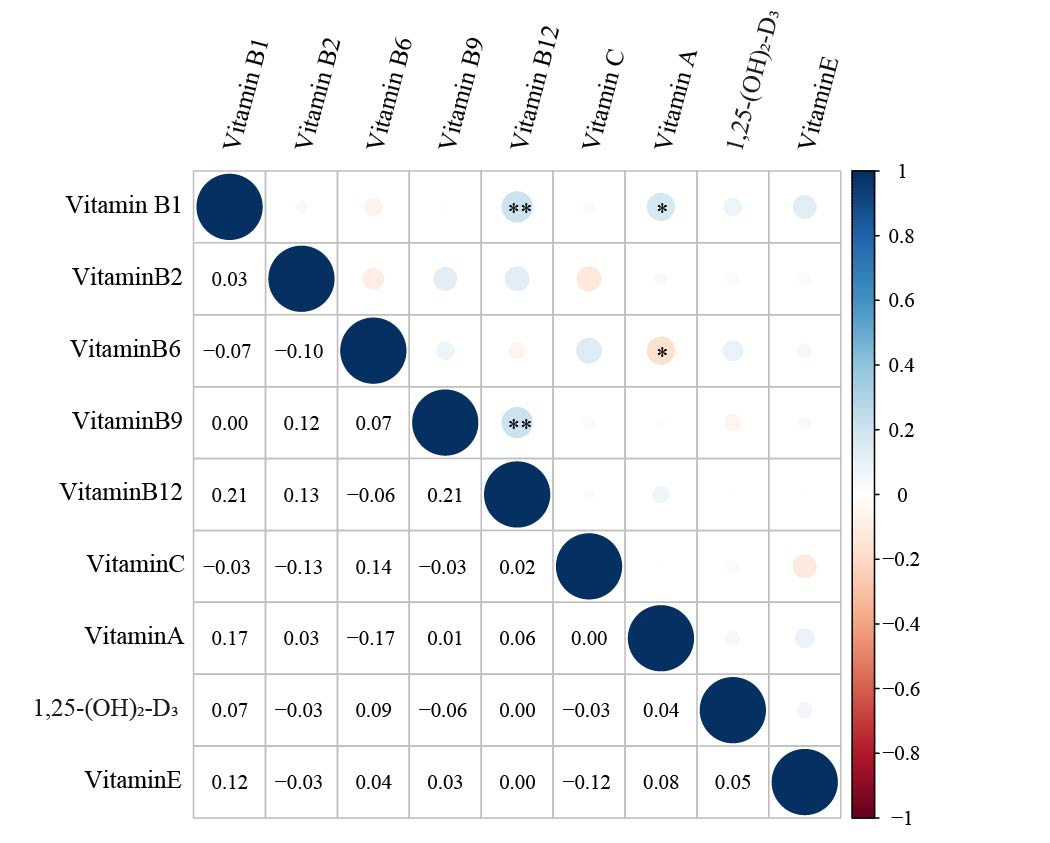


**Figure S3.** Correlation coefficients of Spearman correlation analysis for nine vitamins in serum. ^*^*P*-value ≤ 0.05, ^**^*P*-value ≤ 0.01.


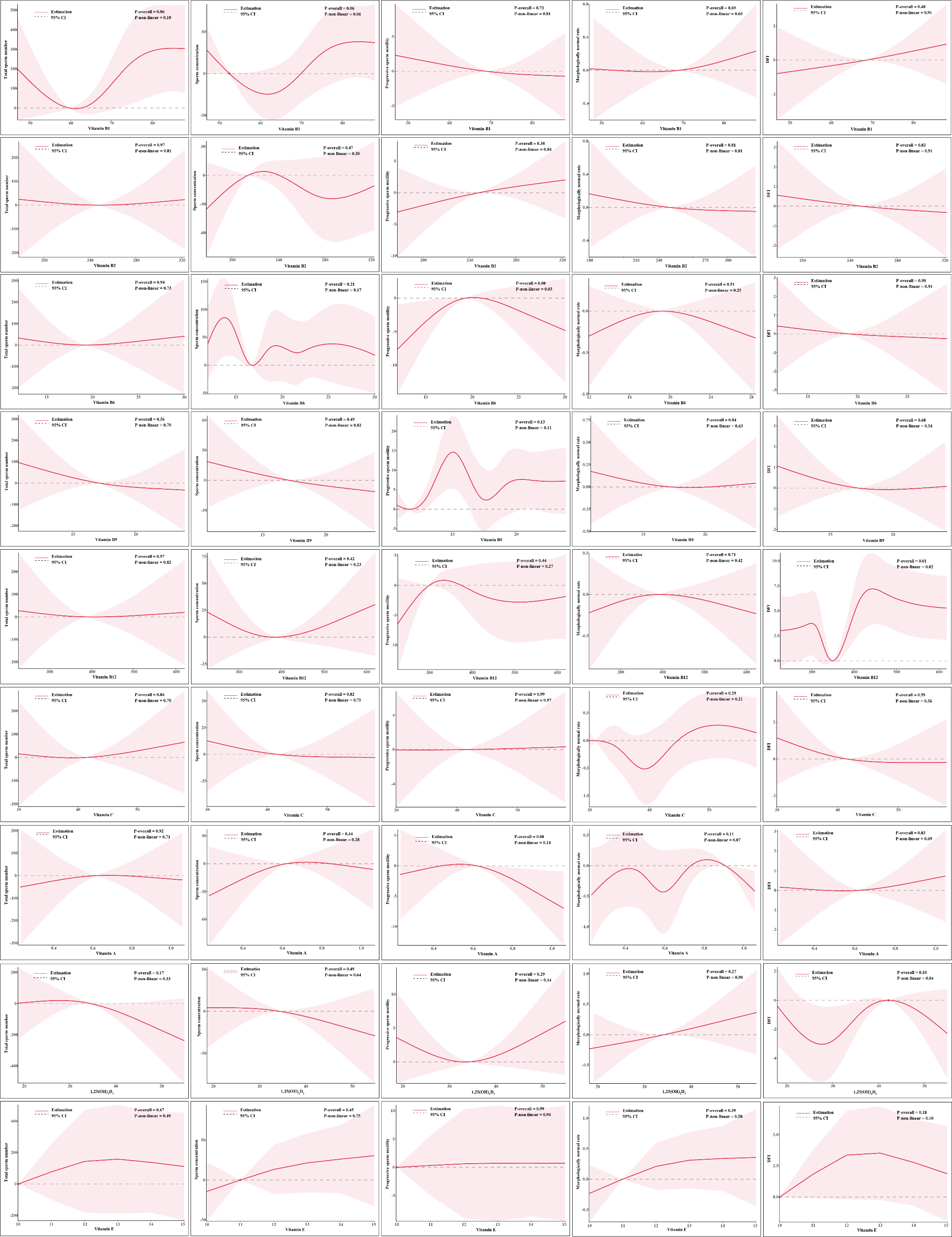


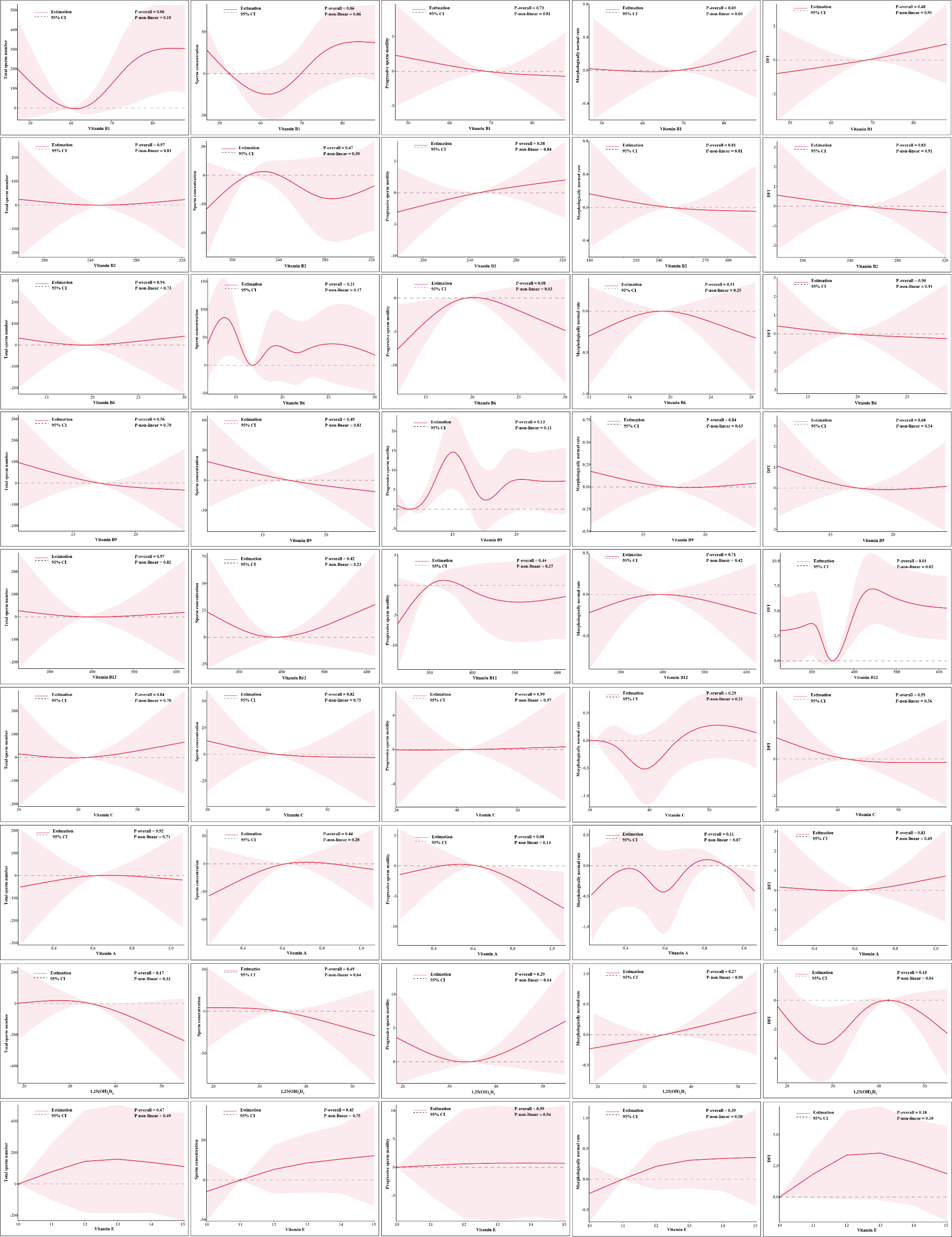


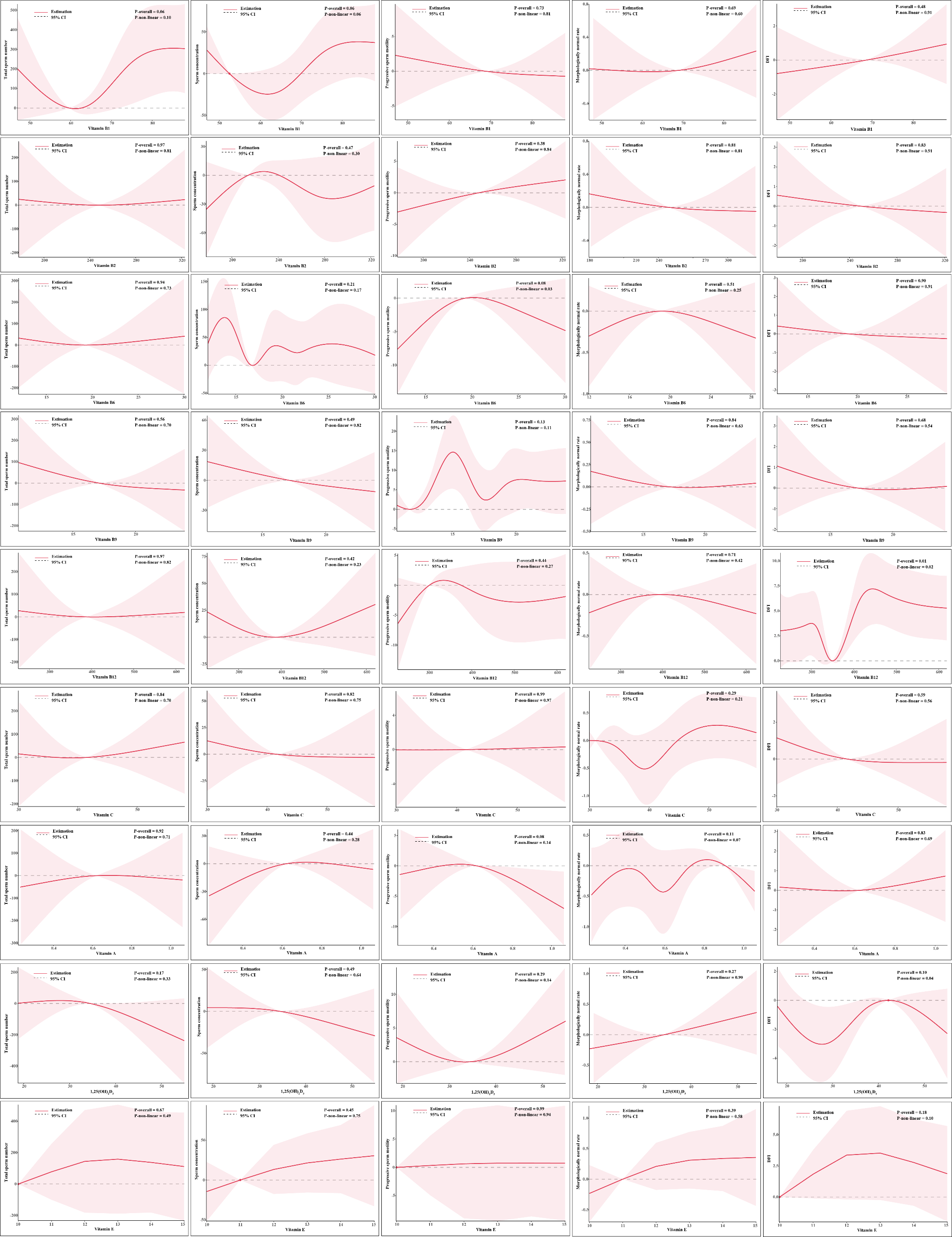


**Figure S4.** The nonlinear associations between vitamins (B1, B2, B6, B9, B12, C, A, D, and E) and sperm parameters (total sperm number, sperm concentration, progressive motile sperm, morphologically normal, and DFI). Models were adjusted for age, BMI, household income, smoke status, and drinking status.

Abbreviations: BMI, body mass index; BKMR, Bayesian kernel machine regression; DFI, DNA fragmetation index.


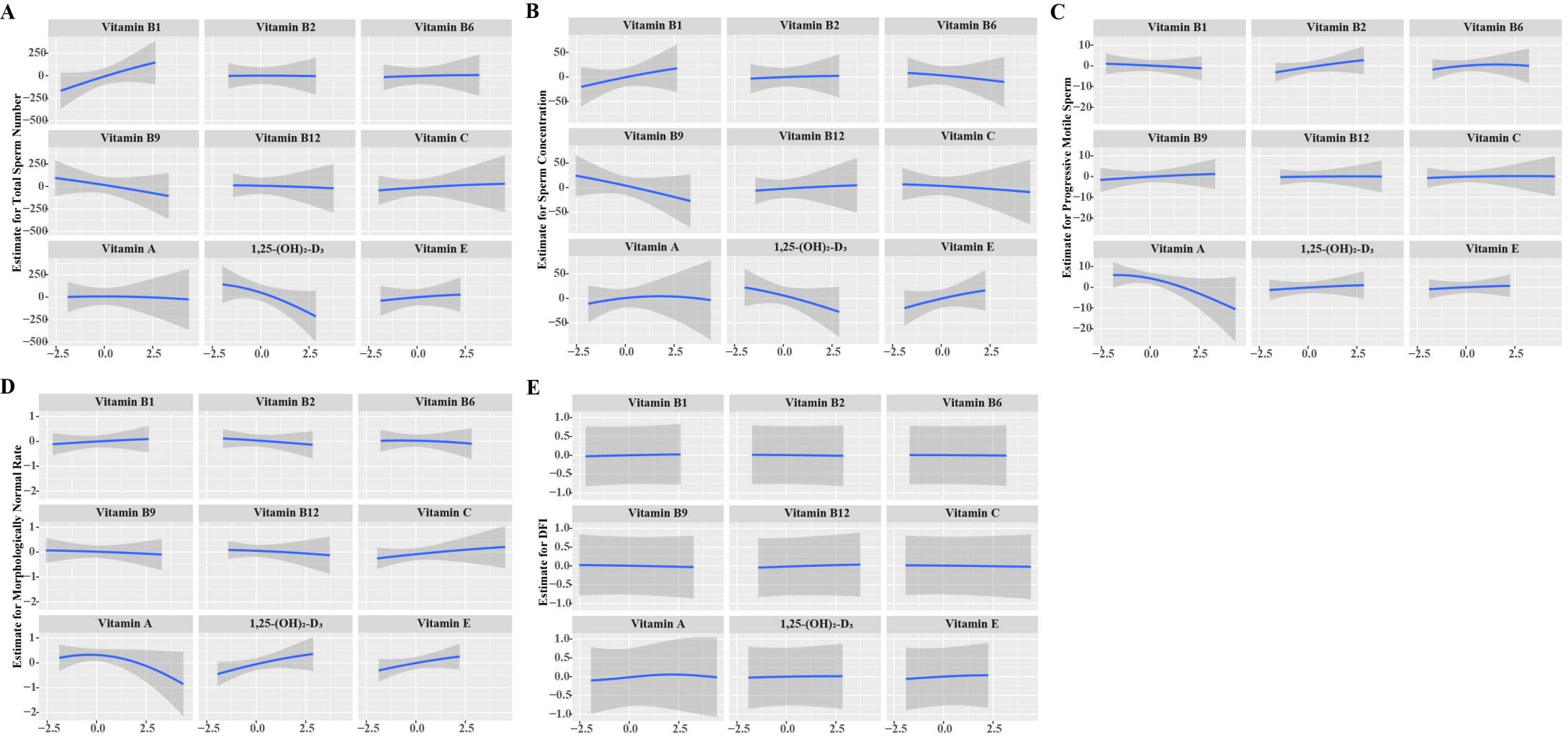


**Figure** S5. Univariate exposure-response relationships and 95% credible intervals between concentrations of individual vitamins and (A) total sperm number, (B) sperm concentration, (C) progressive motile sperm, (D) morphologically normal rate, (E) DFI when fixing other phthalate metabolites at their median values. The BKMR models were adjusted for age, BMI, household income, smoke status, and drinking status.

Abbreviations: BMI, body mass index; BKMR, Bayesian kernel machine regression; DFI, DNA fragmetation index.
